# Pediatric Posttraumatic Cerebral Venous Sinus Thrombosis: Successful Resolution With Rivaroxaban

**DOI:** 10.1155/crpe/8836176

**Published:** 2025-07-27

**Authors:** Yuxuan Zhang, Hui Liu, Hongfang Ding

**Affiliations:** Department of Pediatrics, Shengli Oilfield Central Hospital, Dongying, Shandong, China

**Keywords:** cerebral venous sinus thrombosis, child, traumatic brain injury

## Abstract

Cerebral venous sinus thrombosis (CVST) is rare in children (0.5%–1.0% of pediatric strokes) and uncommonly associated with closed traumatic brain injury. A 7-year-old girl presented with neurological symptoms following a mild closed craniocerebral injury. Early CT imaging revealed subtle findings that were initially overlooked, leading to delayed diagnosis. Subsequent magnetic resonance imaging (MRI) and magnetic resonance venography (MRV) confirmed the diagnosis of CVST. The patient was successfully treated with enoxaparin bridging followed by rivaroxaban, achieving complete thrombus resolution without bleeding complications. This case highlights the diagnostic challenge of posttraumatic CVST in children, where initial imaging signs may be overlooked. It underscores the importance of vigilant imaging interpretation in pediatric brain trauma with persistent symptoms and demonstrates the efficacy and safety of novel oral anticoagulants (NOACs), specifically rivaroxaban, as a therapeutic option in this population.

## 1. Introduction

Cerebral venous sinus thrombosis (CVST) in children is a rare but potentially devastating condition, accounting for approximately 0.5%–1.0% of all strokes [1]. Its incidence is particularly low in children, estimated at about 7 cases per million [2]. CVST is even rarer after closed traumatic brain injury [3]. Diagnostic complexity stems from nonspecific symptoms (e.g., headache and vomiting) overlapping with concussion, frequently delaying neuroimaging confirmation. Current guidelines recommend D-dimer screening and neuroimaging for suspected cases, yet pediatric-specific data on biomarker reliability and optimal anticoagulation remain scarce. Low-molecular-weight heparin (LMWH) is traditionally preferred in the acute stage, followed by a transition to oral warfarin or novel oral anticoagulants (NOACs) based on individual differences [4].

This report presents a 7-year-old girl who developed CVST following a minor household fall, initially misdiagnosed as a concussion due to isolated symptoms and error report of computed tomography (CT) in the community hospital. Her subsequent diagnosis via magnetic resonance imaging (MRI)/magnetic resonance venography (MRV) and successful treatment with anticoagulant therapy, achieving complete recanalization without complications, highlight critical clinical lessons.

## 2. Case Presentation

A 7-year-old girl was admitted to the hospital due to dizziness and vomiting after trauma. Three days before admission, the girl jumped up on the slippery ground at home and accidently slipped, causing dizziness and vomiting. Fifteen hours after the fall, the child experienced repeated episodes of vomiting and persistent dizziness, prompting a visit to a community hospital. The patient was evaluated and underwent a CT scan of the head, which did not reveal any abnormalities. She was diagnosed with a concussion and managed conservatively with fluid replacement and rest. However, her headaches and vomiting persisted for up to 60 h following initial treatment, showing no significant relief.

**Figure 1:**
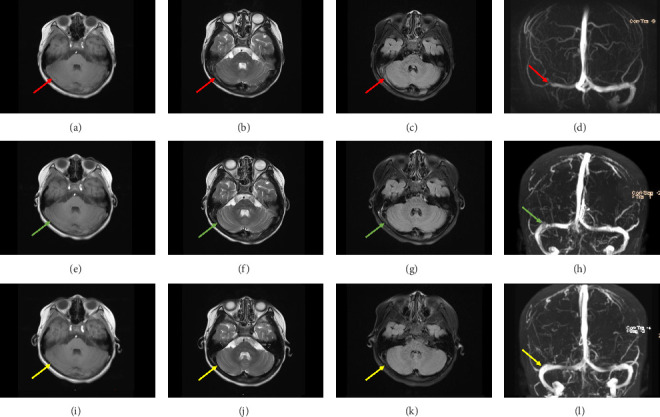
Dynamic changes in MRI/MRV images of patients before and after treatment. Pretreatment (a–d): (a) Axial T1-weighted image (T1WI) showing mixed signals, predominantly isointense and hyperintense, in the right transverse sinus (red arrow). (b) Axial T2-weighted image (T2WI) showing high signal intensity in the right transverse sinus (red arrow). (c) Axial T2 FLAIR image showing high signal intensity in the right transverse sinus (red arrow). (d) Coronal MRV image showing thrombosis in the right transverse sinus and sigmoid sinus, with loss of flow void (red arrow). 7 Days posttreatment (e–h): (e) Axial T1WI showing mixed signals, predominantly isointense and hyperintense, in the right transverse sinus (green arrow). (f) Axial T2WI showing partial recanalization of the right transverse sinus, with T2WI returning to low signal intensity (green arrow). (g) Axial T2 FLAIR image showing partial recanalization of the right transverse sinus, with FLAIR returning to low signal intensity (green arrow). (h) Coronal MRV image showing partial restoration of flow void in the right transverse sinus and sigmoid sinus (green arrow). 3 Months Posttreatment (i–l): (i) Axial T1WI showing no significant abnormal signal in the right transverse sinus (yellow arrow). (j) Axial T2WI showing patency of the right transverse sinus, with low signal intensity (yellow arrow). (k) Axial T2 FLAIR image showing patency of the right transverse sinus, with low signal intensity (yellow arrow). (l) Coronal MRV image showing normal flow void in the right transverse sinus and sigmoid sinus (yellow arrow).

Given the lack of clinical improvement, the child was brought to our institution for further evaluation. Preliminary assessment showed stable vital signs (including normotension) and a Glasgow Coma Scale score of 15. The neurological examination showed no abnormalities, with equal and reactive pupils, and no optic disc edema. Laboratory tests (complete blood count, C-reactive protein, procalcitonin, and coagulation function) including D-dimer were within normal limits. Considering the persistent and progressive symptoms, a more advanced neuroimaging was deemed necessary. A head MRI along with MRV was performed, revealing thrombosis in the right transverse sinus, sigmoid sinus, and intracranial segment of the internal jugular vein (Figures 1(a), 1(b), 1(c), and 1(d)).

Following the diagnosis of CVST, the patient was started on a regimen of subcutaneous enoxaparin sodium (25 mg/2500 IU every 12 h), with gradual resolution of dizziness and vomiting. After a week of hospitalization and clinical stabilization, followed-up MRV showed a significant reduction in thrombus burden, with visible contrast in the right internal jugular vein, transverse sinus, and sigmoid sinus (Figures 1(e), 1(f), 1(g), and 1(h)). She was discharged on oral rivaroxaban (5 mg twice daily) for a planned 3-month anticoagulation therapy. Over the subsequent 3-month outpatient visits, monthly complete blood count, coagulation parameters, and renal/liver function tests revealed consistently normal results. Follow-up head MRI at 3 months confirmed complete resolution of thrombosis in the right transverse sinus, sigmoid sinus, and intracranial segment of the internal jugular vein. Coagulation parameters remained within normal limits, and no residual symptoms or long-term sequelae were observed, indicating a favorable therapeutic outcome (Figures 1(i), 1(j), 1(k), and 1(l)).

**Figure 2:**
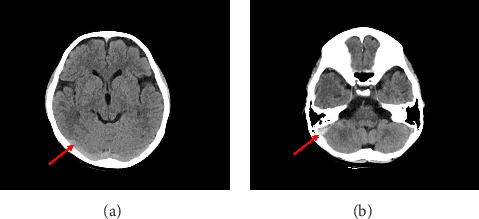
The CT images of patients after onset (a, b). Axial CT images 60 h after onset, showing increased attenuation intensity in the right transverse sinus and sigmoid sinus (red arrows), with no significant brain parenchymal abnormalities.

After the event, we reviewed the initial CT images to look for clues and observed that there were hyperdense signals noted in the right transverse and sigmoid sinuses, indicative of early signs of venous sinus thrombosis (Figure 2), which were unfortunately overlooked during the initial interpretation.

## 3. Discussion

CVST is a rare and severe cerebrovascular disease that exhibits distinct pediatric–adult differences in risk profiles, clinical manifestations, and outcomes [5]. Pediatric CVST is predominantly associated with infections (e.g., otitis media), dehydration, minor trauma, and inherited thrombophilia [6], whereas adult cases are frequently driven by hormonal factors (oral contraceptives, pregnancy, and puerperium) [7], malignancy [8], autoimmune disorders [9], or prothrombotic disorders. Children often present with nonspecific symptoms (e.g., dizziness, persistent vomiting, and lethargy) mimicking benign conditions such as concussion, which may delay diagnosis [10]. In contrast, focal deficits (hemiparesis) or intracranial hypertension (papilledema) are more common in adults [4]. On imaging, CVST mainly involves the superior sagittal sinus/transverse sinus in both adults and children. However, children often demonstrate more extensive sinus involvement and a higher incidence of parenchymal changes, such as hemorrhagic infarcts or edema. Notably, diagnostic accuracy in children may be compromised by skull-related imaging artifacts. [11]. Prognostically, more than 80% pediatric CVST cases can achieve recanalization with early anticoagulation, yet children face higher risks of long-term neurodevelopmental sequelae (cognitive deficits and epilepsy) compared to adults' generally favorable recovery [12]. The differences between children and adults necessitate that clinicians must recognize subtle pediatric presentations, particularly posttraumatic symptoms persisting beyond 48 h, to mitigate diagnostic delays.

The association between minor head trauma and pediatric CVST may involve synergistic effects of endothelial injury [13], hemodynamic alterations [14], and developmental vascular vulnerability. Mechanical forces from low-impact trauma could disrupt venous sinus endothelium, triggering tissue factor release and platelet activation [13]. Pediatric venous systems, with thinner walls and lower baseline blood flow velocity, are prone to shear stress–induced thrombosis even following minor trauma. Concurrently, trauma-induced local inflammation may upregulate prothrombotic cytokines (e.g., IL-6) while suppressing anticoagulant pathways [15]. Hemodynamic stasis due to perivascular edema could further amplify thrombus formation, aligning with the delayed symptom onset (15–72 h posttrauma) observed here [14]. Notably, this contrasts with adult trauma-related CVST, which typically requires direct sinus injury (e.g., skull fractures) or preexisting hypercoagulable states. In children, the developmental immaturity of fibrinolysis and endothelial repair mechanisms may act as a “second hit” enabling thrombosis without traditional risk factors. This case highlights a potential pediatric-specific “two-hit” model: mechanical endothelial disruption caused by minor trauma occurring on a background of age-dependent vascular fragility. In addition, studies have shown that genetic variations in genes, such as *SCN1A*, *MTHFR*, and *PROS1*, may increase susceptibility to CVST [16–18]. As a multifactorial disease, although the patient in this case has no medical history or family history, the occurrence of CVST cannot completely rule out the influence of genetic factors. Unfortunately, we were unable to obtain genetic testing data for this patient.

The therapeutic management of pediatric CVST continues to evolve, with ongoing debate regarding the optimal anticoagulation strategy. While conventional agents (LMWH/warfarin) have well-established safety profiles and long-term clinical experience, the emergence of NOACs offers notable advantages in terms of convenience, predictable pharmacokinetics, and reduced monitoring requirements. Current guidelines predominantly recommend conventional anticoagulation as first-line therapy [19]. This case illustrates a successful transition strategy: After 7 days of subcutaneous enoxaparin, the patient received 3 months of oral rivaroxaban. Follow-up imaging confirmed complete thrombus resolution without bleeding complications or neurological deficits. This outcome is consistent with recent clinical trials and meta-analyses showing that NOACs are noninferior to traditional anticoagulation regimens in terms of efficacy, with potentially lower rates of minor bleeding events [20–22]. While concerns remain about child-specific NOAC dosing due to limited pharmacokinetic data in pediatric populations, this case provides pragmatic evidence supporting their judicious use: The normalization of coagulation profiles and serial imaging–confirmed thrombus resolution aligns with the ASH guidelines permitting NOACs in selected patients. This outcome underscores NOACs' viability for uncomplicated pediatric CVST. Further prospective studies are needed to establish age-specific dosing recommendations and long-term safety profiles.

This case highlights the importance of recognizing subtle imaging findings and maintaining a high index of suspicion for CVST in pediatric patients presenting with persistent headache and vomiting following head trauma, even if the initial CT report indicated no abnormalities. Notably, a normal D-dimer level (observed here at 60 h posttrauma) cannot exclude CVST due to potential late testing after thrombus stabilization. The successful use of rivaroxaban demonstrates the efficacy and safety of NOACs in this population while underscoring that early anticoagulation is crucial to prevent irreversible complications such as epilepsy. Furthermore, this patient's atypical presentation reinforces that developmental venous fragility in children lowers the threshold for thrombosis, suggesting that active imaging examinations and careful image interpretation should be carried out in prolonged postconcussive syndromes.

## Data Availability

The data supporting this report's conclusions are included within the report. Other nonrelevant patient data are safeguarded under patient privacy regulations and policies.
